# Prognostic scores in primary biliary cholangitis

**DOI:** 10.2144/fsoa-2023-0203

**Published:** 2024-05-14

**Authors:** Ramzi Tababi, Soumaya Mrabet, Imen Akkari, Raida Harbi, Elhem Ben Jazia

**Affiliations:** 1 Department of Gastroenterology, Farhat Hached University Hospital, Sousse, 4000, Tunisia

**Keywords:** predictive scores, primary biliary cholangitis, prognosis, treatment response, ursodeoxycholic acid

## Abstract

**Aim:** Evaluating prognostic scores' utility in predicting ursodeoxycholic acid (UDCA) biochemical response (BR) and long-term liver-related complications in primary biliary cholangitis (PBC) patients. **Patients & methods:** This retrospective single-center study included 50 predominantly female PBC patients (median age: 56) on UDCA treatment. BR was defined by Paris II criteria. Liver-related complications during a median 76-month follow-up were assessed. APRI, ALBI, Mayo, GLOBE and UK-PBC scores were calculated. **Results:** 64% achieved BR, while 40% experienced liver-related complications. All scores showed good BR prediction (concordance statistics: 0.76–0.94) and excellent negative predictive values for 5-year liver complications (concordance statistics: 0.73–0.89). **Conclusion:** Implementing these scores in clinical practice is encouraged due to their effectiveness in predicting BR- and liver-related events.

## Tweetable abstract

Prognostic scores in PBC patients on UDCA (APRI, ALBI, Mayo, GLOBE, UK-PBC), good predictors of treatment response and liver-related events. Their clinical use is encouraged. #PBC #MedicalResearch #Prognosis #UDCA.

## Abstract (plain language summary)

Primary biliary cholangitis (PBC) is a chronic liver disease that requires reliable prognostic tools. This retrospective study of 50 PBC patients assessed prognostic scores' effectiveness in predicting biochemical response (BR) to ursodeoxycholic acid (UDCA) and liver-related complications.

BR defined by Paris II criteria was achieved in 64%, while 40% developed liver-related complications during a 76-month follow-up.

Non-responders to UDCA treatment had an 18-fold increase in the risk of long-term liver complications.

APRI, ALBI, Mayo, GLOBE and UK-PBC scores showed good diagnostic performance for predicting BR (AUROC: 0.76–0.94), with GLOBE and UK-PBC demonstrating relative superiority.

Specific cutoff points (APRI: 0.73, ALBI: -2.24, Mayo: 4.5, GLOBE: 1.26, UK-PBC: 0.0980) effectively identified UDCA treatment responders with high positive predictive values (88–96%).

These scores also predicted 5-year liver-related events with AUROC values ranging from 0.73 to 0.89.

Specific cutoff points (APRI: 0.93, ALBI: -2.07, Mayo: 4.5, GLOBE: 0.62, UK-PBC: 0.098) effectively ruled out PBC patients at low risk of long-term liver-related events, with negative predictive values from 89 to 100%.

Mayo risk score demonstrated superiority in anticipating BR among pretreatment scores, and in predicting liver-related complications.

The ALBI grades effectively stratified patients into distinct prognosis groups.

We recommend using these prognostic scores in clinical practice to predict BR and assess the risk of liver-related events.

Primary biliary cholangitis (PBC) is a chronic autoimmune cholestatic liver disease that affects predominantly females over 40 years of age [1]. At present, the incidence and prevalence of PBC appear to have risen, affecting a greater number of men than previously thought, with an estimated ratio of four to six women for every affected man [2]. While still considered rare, its prevalence has not exceeded 50 per 100,000 persons in various study populations [3]. PBC is characterized by the inflammation and gradual destruction of interlobular bile ducts, ultimately leading to end-stage liver disease and related complications. The disease prognosis has significantly improved since the introduction of ursodeoxycholic acid (UDCA), which remains the first-line treatment for the disease. UDCA has shown to improve histology and survival without liver transplantation (LT) [4]. Assessment of the biochemical response (BR) to treatment is usually performed after 6–12 months, using criteria such as Paris II, which are the most widely used in clinical practice [5]. While UDCA has been the mainstay treatment, other options, such as obeticholic acid, have shown efficacy and gained approval as second-line therapy [1].

However, the disease course may vary among patients, potentially placing some individuals at higher risk of adverse outcomes. BR has been established as a major determinant in disease prognosis [5]. Besides, several baseline laboratory findings, such as aspartate aminotransferase to platelet ratio index (APRI) [6], as well as albumin and bilirubin serum levels [7], have been considered as prognostic factors. Recently, quantitative scores have been developed, including the Global Assessment of Liver Outcomes score for PBC (GLOBE) [8] and UK Primary Biliary Cholangitis score (UK-PBC) [9]. These scores are derived from biological parameters at diagnosis and after treatment, serving as tools to predict survival and end-stage liver disease.

The implication is that scoring systems integrate multiple predictive factors, facilitating the identification of high-risk groups, which in turn could help optimize therapeutic strategies and potentially improve long-term outcomes. This study aimed to assess the performance of prognostic scores in predicting treatment response and long-term liver-related complications in patients with PBC.

## Materials & methods

### Study design

**Figure 1.**
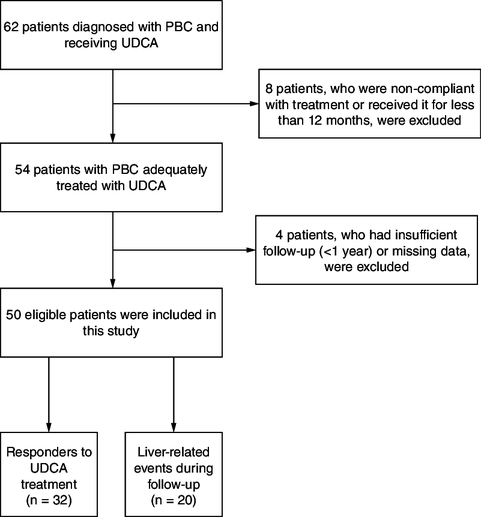
Flowchart of patient selection and outcomes. PBC: Primary biliary cholangitis; UDCA: Ursodeoxycholic acid.

This is a retrospective single-center study, including both inpatients and outpatients diagnosed with PBC, based on clinical practice guidelines [1,10], and who received UDCA treatment for more than 12 months. Patients who were lost to follow-up or did not comply with treatment were excluded. Additionally, patients diagnosed with other liver diseases, except for autoimmune hepatitis, were also excluded from the study (Figure 1).

### Data collection & definitions

Patients' demographic, clinical, biological and therapeutic characteristics were obtained from medical records. The following serum laboratory findings were documented: platelet count (PLT), aspartate aminotransferase (AST), alanine aminotransferase (ALT), gamma-glutamyl transpeptidase (GGT), alkaline phosphatase (ALP), total bilirubin (TB), albumin (Alb) and prothrombin time (PT).

Treatment response to UDCA was defined based on Paris II criteria [5]. Adverse outcomes were defined as the occurrence of liver-related complications during the follow-up period, including ascites, variceal hemorrhage, hepatic encephalopathy and hepatocellular carcinoma (HCC).

### Scores calculation

The scores calculated at baseline were APRI, albumin-bilirubin score (ALBI) and Mayo risk score. Whereas GLOBE and UK-PBC risk scores required post-treatment parameters, hence determined at 12 months of UDCA treatment. The formulas used for calculating each score were as follows:

APRI=((AST  times  the  upper  limit of  normal  (ULN))PLT (109L))×100.

ALBI  score=(log10 TB (μmolL) × 0.66) + (Alb (gL) × −0.085).

Mayo risk score was calculated as the sum of its following subscores:

Age (years) = 0 (<38); 1 (38−62); 2 (≥63).

TB (mgdl) = 0 (<1); 1 (1−1.7); 2 (1.7−6.4); 3 (≥6.4).

Alb (gdl) = 0 (>4.1); 1 (2.8−4.1); 2 (<2.8).

PT = 1 (normal); 2 (prolonged).

Oedema = 0 (absent); 1 (present or controlled with diuretics).

GLOBE score [8] = (0.044378 × age  at start of UDCA therapy + 0.93982 × Ln (TB times the ULN at 1 year follow-up))+ (0.335648 × Ln (ALP times the ULN at 1 year follow-up))− 2.266708 × Alb  level  times  the lower limit of  normal (LLN) at 1 year follow-up−0.002581 × PLT (109L) at 1year follow-up+1.216865.

$$\begin{array}{l} \text{UK}-\text{PBC }\;\text{risk}\;\text{score}\;[9]\;=\;\\ 1-\text{baseline}\;\text{survival}\;\text{function}\;\wedge \exp\;(0.0287854\;\;\times \;(\text{ALP}\;\text{at}\;12\;\text{months}\;\text{times}\;\text{the}\;\text{ULN}\;-\;1.722136304)\\ -\;0.0422873\;\;\times \;\left(\;\left({\left(\frac{\text{ALT}\;\text{or}\;\text{AST}\;\text{at}\;12\;\text{months}\;\text{times}\;\text{the}\;\text{ULN}}{10}\right)}^{-1}\right)-8.675729006\right)\\ +\;1.4199\;\times \;\left(\ln\left(\frac{\text{TB}\;\text{at}\;12\;\text{months}\;\text{times}\;\text{the}\;\text{ULN}}{10}\right)\;+2.709607778\right)\\ -\;1.960303\;\times \;(\text{Alb}\;\text{at}\;\text{baseline}\;\text{times}\;\text{the}\;\text{LLN}\;-\;1.17673001)\\ -\;0.4161954\;\times \;(\text{PLT}\;\text{at}\;\text{baseline}\;\text{times}\;\text{the LLN }-\;1.873564875)). \end{array}$$

Note: for UK-PBC risk score, baseline survivor function = 0.982 (at 5 years); 0.941 (at 10 years); 0.893 (at 15 years).

### Statistical analysis

Categorical variables were presented as absolute and relative frequencies. The chi-square test was used to compare nominal variables. The normality of continuous variables was assessed using the Shapiro–Wilk test. Normally-distributed variables were expressed as means ± standard deviations (SD), while non-normally distributed variables were presented as medians and interquartile ranges (IQR). Independent groups were compared using *t*-test for means and Mann–Whitney *U* test for medians.

Liver event-free survival was estimated using the Kaplan–Meier model, and the Log-rank test was used to compare survival curves. Cox proportional hazards analysis was performed to determine hazard ratios (HR). The prognostic scores' performance in predicting treatment response and the development of liver-related complications over 5 years was evaluated using receiver-operating characteristic (ROC) curves, and the area under the ROC curve (AUROC) was calculated. The optimal cut-off points were identified using Youden's index.

For all statistical tests, a significance level of 5% (p < 0.05) was set. Statistical analyses were performed using IBM SPSS Statistics software (version 26) and GraphPad Prism (version 9) for Windows.

## Results

### Study population

In total, 50 patients with PBC were included in this study (Figure 1). The median age at diagnosis was 56 years (IQR = 19), and the majority of patients were females (n = 49, 98%). Ten patients (20%) had an overlap syndrome associating PBC and autoimmune hepatitis (AIH). Nineteen patients (38%) had cirrhosis at the time of diagnosis, with ten of them (52.6%) being in the decompensated stage. Patients' characteristics, as well as the documented lab findings, are summarized in Table 1.

**Table 1.** Patients' characteristics and pre-treatment lab data.

Parameter		All patients (n = 50)
Age at diagnosis (years)	Median (IQR)	56 (18)
Females/males	n (%)	49 (98)/1 (2)
Associated AIH	n (%)	10 (20)
Cirrhosis at diagnosis	n (%)	19 (38)
Decompensated cirrhosis at diagnosis	n (%)	10 (52.6% of cirrhosis)
PLT (10∧9/l)	Mean ± SD	194.755 ± 95.815
AST (UI/l)	Median (IQR)	58 (50)
ALT (UI/l)	Median (IQR)	49 (45)
GGT (UI/l)	Median (IQR)	152 (248)
ALP (UI/l)	Median (IQR)	298 (368)
TB (μmol/l)	Median (IQR)	12 (15)
Alb (g/l)	Mean ± SD	34.3 ± 8.2
PT (%)	Median (IQR)	91 (23)
Treatment responders	n (%)	32 (64)
Follow-up (months)	Median (IQR)	76 (80)
Liver cirrhosis complications	n (%)	20 (40%)

AIH: Autoimmune hepatitis; Alb: Albumin; ALP: Alkaline phosphatase; ALT: Alanine aminotransferase; AST: Aaspartate aminotransferase; GGT: Gamma-glutamyl transferase; IQR: Inter quartile range; PLT: Platelet count; PT: Prothrombin time; TB: Total bilirubin.

At 12 months of UDCA treatment, 32 patients (64%) were considered responders, while 18 (36%) were classified as non-responders. Table 2 presents the differences in patients' characteristics and pre-treatment lab data between responders and non-responders.

**Table 2.** Differences in patients' characteristics and pre-treatment lab data between responders and non-responders.

Parameter		Responders (n = 32, 64%)	Non-responders (n = 18, 36%)	p-value
Age at diagnosis <50 years	n (%)	10 (31.3)	5 (27.8)	0.797
Females/males	n (%)	32 (100)/0	17 (94.4)/1 (5.6)	0.360
Associated AIH	n (%)	7 (21.9)	3 (16.7)	0.479
Cirrhosis at diagnosis	n (%)	5 (15.6)	14 (77.8)	<0.001
PLT (10∧9/l)	Mean ± SD	210.580 ± 79.308	167.500 ± 116.528	0.175
AST (UI/l)	Median (IQR)	50 (46)	66.5 (82)	0.199
ALT (UI/l)	Median (IQR)	43 (50)	55.5 (46)	0.363
GGT (UI/l)	Median (IQR)	150 (217)	195 (371)	0.640
ALP (UI/l)	Median (IQR)	285 (291)	413 (488)	0.097
TB (μmol/l)	Median (IQR)	10 (8)	34.4 (52)	**<0.001**
Alb (g/l)	Mean ± SD	36.6 ± 8.3	30.5 ± 6.6	**0.009**
PT (%)	Median (IQR)	95.5 (14)	77.5 (30)	**0.003**

Compared with non-responders, responders to treatment had significantly higher baseline prothrombin time and albumin level and lower bilirubin level, as indicated with the bold values.

Alb: Albumin; AIH: Autoimmune hepatitis; ALT: Alanine aminotransferase; ALP: Alkaline phosphatase; AST: Aspartate aminotransferase; GGT: Gamma-glutamyl transferase; IQR: Interquartile range; PLT: Platelet count; PT: Prothrombin time; TB: Total bilirubin.

The median follow-up duration was 76 months (IQR = 80). Among the patients, 20 (40%) experienced at least one liver-related complication, with a median time of 72 months (IQR = 77) for the first complication to arise. Variceal hemorrhage occurred in nine patients (45%), ascites in eight patients (40%), hepatic encephalopathy in one patient (5%) and HCC in two patients (10%).

The median (IQR) values of the calculated scores were as follows: 0.91 (0.79) for APRI, 4 (2) for the Mayo risk score, 0.4 (2.48) for the GLOBE score, -2.171 (1.184) for ALBI score, 0.0420 (0.1485) for the UK-PBC risk score at 5 years, 0.1338 (0.4000) for the UK-PBC risk score at 10 years, and 0.2345 (0.5787) for the UK-PBC risk score at 15 years.

### Predicting adverse outcomes by biochemical response

**Figure 2.**
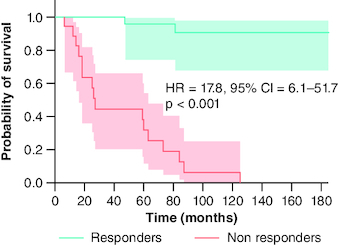
Liver event-free survival analysis by treatment response, using Kaplan–Meier model and Log-rank test, for PBC patients treated with UDCA. HR: Hazard ratio; PBC: Primary biliary cholangitis; UDCA: Ursodeoxycholic acid.

Paris II criteria could significantly distinguish survival distribution based on long-term outcomes, with a HR value of 17.8 (95% confidence interval [CI]: 6.1–51.7, p < 0.001) in non-responders. Median survival time without liver-related complications (in months) was significantly higher in responders (185, 95% CI: 168–202) compared with non-responders (27, 95% CI: 23–31; p < 0.001). Survival rates without adverse outcomes at 5 and 10 years of follow-up were 96 and 91%, respectively, in responders, whereas they were 32 and 0% in non-responders (Figure 2).

### Scores predicting treatment response

**Figure 3.**
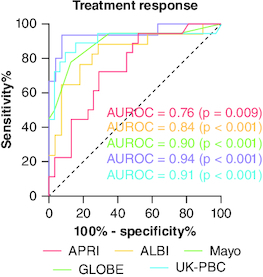
ROC curves assessing the discriminatory performance of prognostic scores in predicting biochemical response for primary biliary cholangitis patients treated with ursodeoxycholic acid. ALBI: Albumin-bilirubin score; APRI: Aspartate aminotransferase to platelet ratio index; AUROC: Area under the curve; BR: Biochemical response; GLOBE: Global Assessment of Liver Outcomes score; PBC: Primary biliary cholangitis; ROC: Receiver operating characteristic; UDCA: Ursodeoxycholic acid; UK-PBC: UK Primary Biliary Cholangitis score.

All APRI, ALBI, Mayo, GLOBE and UK-PBC risk scores indicated treatment response at 12 months (Figure 3). APRI was a modestly significant predictor of treatment response with an AUROC of 0.76 (p = 0.009). A score value of 0.73 or lower had a specificity and positive predictive value (PPV) of 89%, but it showed poor sensitivity (52%) and negative predictive value (NPV) (52%), resulting in an overall diagnostic accuracy (DA) of 65%.

The ALBI score proved to be a better predictor of response, with an AUROC of 0.84 (p < 0.001). Using an optimal cutoff of -2.24, the sensitivity, specificity, PPV, NPV and DA were 71, 88, 91, 65 and 78%, respectively.

The Mayo risk score exhibited an AUROC of 0.90 (p < 0.001). A score value below 4.5 showed a sensitivity of 88%, specificity of 78%, PPV of 88%, NPV of 78% and an accuracy of 84%.

Both GLOBE score and UK-PBC risk scores demonstrated the highest performance in indicating response according to Paris II criteria, with respective AUROC values of 0.94 and 0.91 (both p < 0.001). A GLOBE score under 1.26 demonstrated 93% sensitivity, 93% specificity, 96% PPV, 88% NPV and 93% accuracy. For the UK-PBC risk score (at 5 years), a value lower than 0.0980 had an 84% sensitivity, 89% specificity, 93% PPV, 76% NPV and 86% DA.

### Scores predicting outcomes

**Figure 4.**
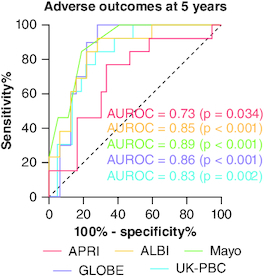
ROC curves assessing the discriminatory performance of prognostic scores in predicting liver-related events over 5 years, for primary biliary cholangitis patients treated with ursodeoxycholic acid. ALBI: Albumin-bilirubin score; APRI: Aspartate aminotransferase to platelet ratio index; AUROC: Area under the curve; GLOBE: Global Assessment of Liver Outcomes score; PBC: Primary biliary cholangitis; ROC: Receiver operating characteristic; UDCA: Ursodeoxycholic acid; UK-PBC: UK Primary Biliary Cholangitis score.

**Figure 5.**
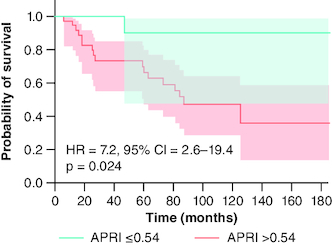
Liver event-free survival analysis by APRI 0.54 threshold, using Kaplan–Meier model and Log-rank test, for primary biliary cholangitis patients treated with ursodeoxycholic acid. APRI: Aspartate aminotransferase to platelet ratio index; HR: Hazard ratio; PBC: Primary biliary cholangitis; UDCA: Ursodeoxycholic acid.

The calculated scores were all predictive of adverse outcomes as well (Figure 4). APRI had a moderate performance in predicting liver-related events over 5 years with an AUROC of 0.73 (p = 0.034). The sensitivity (77%) and NPV (89%) at the cutoff of 0.93 were better than its specificity (67%) and PPV (45%), resulting in an accuracy of 69%. Patients with a score higher than 0.54 developed a liver event earlier than others, with a median time-to-onset of 87 months (95% CI = 46–128 months) compared with 186 months (95% CI = 157–214 months) for those with lower scores (p = 0.024) (Figure 5).

**Figure 6.**
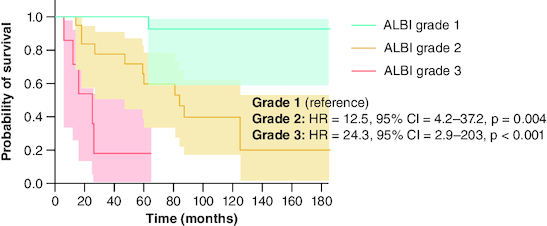
Liver event-free survival analysis by ALBI grades, using Kaplan–Meier model and log-rank test, for primary biliary cholangitis patients treated with ursodeoxycholic acid. ALBI: Albumin-bilirubin score; CI: Confidence interval; HR: Hazard ratio; PBC: Primary biliary cholangitis; UDCA: Ursodeoxycholic acid.

The AUROC of the ALBI score for predicting liver events in 5 years was 0.85 (p < 0.001). A score ≥-2.07 had corresponding sensitivity, specificity, PPV, NPV and DA values of 92, 72, 57, 96 and 78%, respectively. Median liver event-free survival was significantly different (all p < 0.001) among ALBI grade 1 (191 months, 95% CI: 173–210), grade 2 (84 months, 95% CI: 51–117), and grade 3 (25 months, 95% CI: 60–190). The respective survival rates at 5 years were 93, 60 and 19%, and at 10 years, it was 93% for grade 1, 40% for grade 2, and not defined for grade 3 due to 0 patients at risk (Figure 6).

The Mayo risk score had an AUROC of 0.89 (p < 0.001) for predicting outcomes. It was particularly effective in excluding patients at risk of liver complications in 5 years, with an excellent NPV (94%) for scores ≥4.5. Additionally, it showed a sensitivity of 85%, specificity of 82%, PPV of 61% and a DA of 82%.

**Figure 7.**
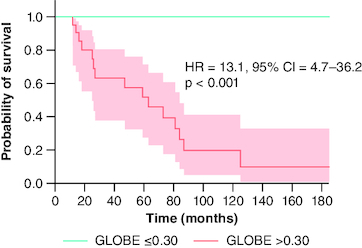
Liver event-free survival analysis by GLOBE score threshold of 0.30, using Kaplan–Meier model and Log-rank test, for primary biliary cholangitis patients treated with ursodeoxycholic acid. GLOBE: Global Assessment of Liver Outcomes score; HR: Hazard ratio; PBC: Primary biliary cholangitis; UDCA: Ursodeoxycholic acid.

The AUROC of the GLOBE score for predicting adverse outcomes was 0.86 (p < 0.001). It demonstrated excellent sensitivity and NPV, correctly identifying patients at risk of experiencing liver events at 5 years, as well as those safe from such risks. A score above 0.62 had 100% sensitivity, 72% specificity, 53% PPV, 100% NPV and 79% DA. Patients with a GLOBE score of 0.30 or greater had significantly decreased event-free survival at 5 and 10 years (52 and 20%, respectively) compared with patients below this threshold (100% for both at 5 and 10 years, p < 0.001) (Figure 7).

The UK-PBC risk score was also predictive of 5-year liver adverse outcomes, with an AUROC of 0.83 (p = 0.002). An optimal cutoff of 0.0980 had a sensitivity of 85%, specificity of 73%, PPV of 52%, NPV of 93% and DA of 76%.

## Discussion

The prognostic scores studied in our research demonstrated moderate to excellent performance in selecting PBC patients receiving UDCA who were more likely to be non-responders to treatment and develop liver-related events.

The importance of biochemical response (BR) as a major prognostic determinant in PBC is widely recognized. Paris II criteria, developed based on a French cohort of 165 early-stage PBC patients by Corpechot *et al.* [5], showed better performance than other criteria (Paris I, Barcelona, Rotterdam, and Rochester II) in identifying high-risk patients for liver events, characterized by progression toward cirrhosis or its complications, LT, and mortality related to liver. Paris II demonstrated the best diagnostic performance, with a great positive likelihood ratio (infinite) and a poor negative likelihood ratio of 0.5, thus lacking accuracy in identifying patients at low risk. Paris I criteria could be more adapted to advanced disease patients [5,11]. In our study, we used Paris II criteria to define BR, even though 38% of our patients were cirrhotic at baseline. We also found these criteria to be predictive of outcomes. In the aforementioned cohort [5], the 5-year survival rates without adverse outcomes were 95% in responders to treatment, and 85% in non-responders. Compared with our study, responders had a similar survival at 5 years (96%), however, non-responders had lower survival (32%), most likely owing to the fact that our study population comprises not only early disease stages, but also cirrhotic patients. A recent Japanese retrospective cohort [12] of 196 early PBC patients found that Paris II criteria were an independent predictor of overall survival (HR: 3.948, 95% CI: 1.293–12.054, p = 0.016) and hepatic mortality (HR: 10.461, 95% CI: 1.231–88.936, p = 0.032) among PBC patients, supporting the prognostic value of BR according to Paris II criteria. Furthermore, Harms *et al.* [13] conducted a large multicenter study of 3,224 PBC patients with various histological disease stages. They showed that Paris II criteria predicted the occurrence of hepatic complications with a HR of 4.654 (95% CI: 3.270–6.622, p < 0.001).

An interesting study published in the year 2020 by Murillo *et al.* [14] including more than 2200 PBC patients with normal bilirubin levels found that an optimal cutoff of 0.6 xULN of bilirubin was a significant predictor of outcomes. Patients with bilirubin ≤0.6 xULN had a 10-year death/LT-free survival of 91.3%, compared with 79.2% (p < 0.001). Besides, 10-year survival rates were 93.2% in patients with normal ALP, and 86.1% in those with ALP levels between 1 and 1.67 xULN. The intriguing findings from this robust study lay the groundwork for a potential redefinition of treatment response that predicts a more favorable prognosis.

The scores we studied are primarily based on biological prognostic markers, including PLT, Alb, TB, ALP and transaminases [15].

APRI, a non-invasive test to assess liver fibrosis, has shown predictive value for BR and risk of liver decompensation in previous studies [6,15]. In a British cohort of 386 patients, Trivedi *et al.* [6] demonstrated that baseline APRI and 12-month treatment APRI could stratify patients at risk using a cutoff of 0.54. Our findings were consistent with these studies, as we observed that patients with higher APRI scores experienced shorter event-free survival, while those with lower APRI scores showed better survival rates. According to Trivedi *et al.* [6], baseline APRI demonstrated an AUROC of 0.78 for predicting LT-free survival, a result comparable to the AUROC in our study, which was 0.73 for predicting liver events. Additionally, the liver event-free survival rate at 5 years reported in this earlier study for patients with a baseline APRI higher than 0.54 was 90% (compared with 80% in patients with lower scores), a finding consistent with our study (90% in patients with APRI >0.54 vs 70% in patients with lower scores). In a study involving 272 PBC patients with different histological stages, Joshita *et al.* [16] confirmed earlier observations, emphasizing the discriminative ability of APRI in predicting liver complications, with an AUROC of 0.79. They also endorsed a consistent threshold of 0.54: patients surpassing this value faced a 5% risk of disease progression and increased liver complications at 5 years (compared with 0%) and a 10% risk at 10 years (compared with 1%). In the previously mentioned study by Harms *et al.* [13], baseline APRI was identified as a significant predictor of liver events, independent of BR, with a score above 0.54 showing a HR of 5.32 (95% CI: 3.82–7.41, p < 0.001). Similarly, in our study, an APRI score higher than 0.54 demonstrated a HR of 7.2 (95% CI: 2.6–19.4, p = 0.024). Conversely, in a recently published study by Feng J *et al.* [17] involving 397 advanced PBC patients, APRI exhibited minimal discriminative performance in predicting liver events, with an AUROC of 0.592 (95% CI: 0.536–0.647).

Although traditionally used to assess liver function in cirrhotic patients with HCC, the ALBI score has been investigated as a prognostic tool in PBC. Our findings align with recent research [18,19] that demonstrated ALBI score's ability to stratify PBC patients at risk of decompensation. In a retrospective Japanese multicenter study of 409 patients with PBC of all stages, Ito *et al.* [18] showed that ALBI had the highest AUROC among studied scores for predicting adverse outcomes (0.94, 0.91 and 0.90 at 3, 5 and 10 years, respectively). LT-free survival rates at 5 years for the three ALBI grades in this study were 98, 80 and 24%, respectively. These rates closely align with the 5-year liver event-free survival rates of ALBI grades found in our study (93, 60 and 19%, respectively). In the study by Chen *et al.* [19], the performance of ALBI was investigated in 219 patients with compensated PBC cirrhosis. They found an AUROC of 0.87 for predicting 5-year liver-related mortality, with 90% sensitivity and 77% specificity at a cutoff value of -1.47. ALBI demonstrated good performance in our study as well, with an AUROC of 0.83, a sensitivity of 92%, and a specificity of 72%, albeit with a lower optimal cutoff (-2.07), possibly due to the inclusion of not only cirrhotic patients in the study population.

The Mayo model proved to be predictive of both BR and adverse outcomes in two randomized controlled trials of UDCA treatment for PBC, conducted in the 90s by Angulo *et al.* [20] and Kilmurry *et al.* [21]. Specifically, patients with a Mayo score above 4.5, calculated at 6 months of treatment, were likely to be non-responders to treatment at 24 months – a threshold similar to ours. Furthermore, Angulo *et al.* [20] found that a Mayo score cutoff value of 4 predicted the development of esophageal varices. They also demonstrated that the Mayo score could stratify patients into different risk groups for overall survival and LT-free survival: low risk (score below 5.37), intermediate risk (score between 5.37 and 6.42), and high risk (score above 6.42). In our study, we identified an optimal cutoff of 4.5 with good discriminative ability for predicting liver event-free survival.

A recent study by Goet *et al.* [22] compared Mayo, GLOBE, and UK-PBC risk scores in predicting outcomes in a large international cohort of 1100 PBC patients. Mayo score was found to be an accurate survival predictive tool, showing high concordance statistics at different time points [0.76 (95% CI: 0.72–0.81)], with no statistical difference compared with other scores. Mayo score exhibited a comparable concordance statistic of 0.702 (95% CI: 0.653–0.751) in another study [17]. Our findings corroborate the performance of Mayo score, indicating an even higher AUROC of 0.89.

GLOBE and UK-PBC risk scores, established from large studies, integrate initial biochemical disease severity and UDCA response, providing valuable tools to predict outcomes. The GLOBE score, developed from an international meta-analysis of 4119 patients [8], identifies non-responders with reduced 5- and 10-year liver transplantation-free survival (78 and 57%, respectively) and responders with comparable survival to the general population (98 and 92%) using a cutoff of 0.30. In our study, we found a significant distribution of liver event-free survival using the GLOBE score, with an optimal cutoff point of 0.62, potentially influenced by the higher proportion of patients over 50 years old (70%). Lammers *et al.* [8] also established different optimal thresholds based on age groups, notably 0.60 for subjects aged between 52 and 58 years and 1.01 for those aged between 58 and 66 years. Recent evidence from the large GLOBAL PBC cohort showed that the change in GLOBE score during the first and second years predicts death/LT-free survival with respective HR of 2.28 (95% CI: 1.81–2.87, p < 0.001) and 2.19 (95% CI: 1.67–2.86, p < 0.001), independently from the baseline score [23]. This suggests monitoring the GLOBE score at different time points to further optimize outcome prediction.

The UK-PBC score, developed by Carbone *et al.* [9], is based on a large cohort of 1916 British patients and further validated in an independent cohort of 1249 patients. This risk score allows for accurate long-term prediction of LT and liver-related death at 5, 10 and 15 years, with an AUROC >0.90. In our study, the UK-PBC score demonstrated good performance in predicting hepatic complications at 5 years, with an AUROC of 0.80.

Recent research by Goet *et al.* [22] and Alomari *et al.* [24] has confirmed the accuracy of GLOBE and UK-PBC scores as prognostic models predictive of LT and mortality. The respective concordance statistics at 1 and 15 years were 0.80 and 0.75 for GLOBE, and 0.74 for UK-PBC (at both 1 and 15 years). These scores have been further validated in a Chinese cohort [25], suggesting an additional role of anti-gp210 as a pejorative prognostic marker to enhance the discriminative ability of these scores. However, a primary limitation of GLOBE and UK-PBC scores is their inability to identify patients less likely to respond to UDCA before treatment initiation, considering that they are calculated at 12 months. To address this issue, a novel score has been developed, namely the UDCA Response Score (URS), which has been validated and proved satisfactory performance [26,27], yet not widely used in practice.

The conclusive discussion underscores the importance of selecting optimal prognostic scores for PBC outcomes. In practical terms, our investigation points to the GLOBE score, calculated at 12 months, as a robust choice for indicating treatment response, while the Mayo risk score stands out for anticipating liver events. Additionally, prior to the commencement of UDCA, the Mayo score proved to be the most solid predictor of treatment response. Furthermore, our findings highlight the utility of classifying patients into one of the three ALBI grades, as effectively stratifying the risk of decompensation. These insights not only enhance the precision of prognostic assessments in PBC but also provide valuable guidance for clinicians in tailoring management strategies based on individual patient profiles.

## Limitations

Our study has several limitations, including the retrospective design, the monocentric nature and the limited number of patients. The heterogeneity of the study population, as per having both early and advanced disease, could potentially impact the general applicability of the findings. Besides, histological disease stage data was only available for a subset of patients. Moreover, the study lacked sufficient cases to evaluate mortality.

## Conclusion

In conclusion, our results display the satisfactory performance of various prognostic scores (APRI, ALBI, Mayo, GLOBE and UK-PBC) in predicting both treatment response to UDCA and long-term liver-related adverse outcomes in PBC patients. Specifically, the GLOBE score emerged as a reliable indicator of treatment response, while the Mayo risk score, along with ALBI grades, accurately stratified the risk of liver-related events. Mayo score was also the optimal pretreatment predictor for UDCA response. We recommend implementing their use in clinical practice, utilizing the suggested thresholds, to help identify patients at risk, who might benefit from close monitoring and, eventually, a more intensive therapy.
